# Metabolic Reprogramming Through Polyphenol Networks: A Systems Approach to Metabolic Inflammation and Insulin Resistance

**DOI:** 10.3390/medsci13030180

**Published:** 2025-09-05

**Authors:** Shakila Jahan Shimu, Jawad Ul Karim Mahir, Fardin Al Fahad Shakib, Arafath Amin Ridoy, Ratin Al Samir, Nadia Jahan, Md Fahim Hasan, Sadman Sazzad, Shamima Akter, Mohammad Sarif Mohiuddin, Md Jalal Ahmed Shawon, Mohammad Hossain Shariare, Mohammad Mohabbulla Mohib, Mohammad Borhan Uddin

**Affiliations:** 1 Department of Health Informatics, Harrisburg University of Science and Technology, 326 Market St., Harrisburg, PA 17101, USA; shakilajahan@hotmail.com; 2 Department of Pharmaceutical Sciences, North South University, Dhaka 1229, Bangladesh; jawad.mahir@northsouth.edu (J.U.K.M.); fardin.shakib@northsouth.edu (F.A.F.S.); arafath.ridoy@northsouth.edu (A.A.R.); ratin.samir@northsouth.edu (R.A.S.); nadiajahan.njn@gmail.com (N.J.); fahim.hasan10@northsouth.edu (M.F.H.); mohammad.shariare@northsouth.edu (M.H.S.); 3 Department of Biomedical Engineering and Informatics, Luddy School of Informatics, Computing and Engineering, Indiana University Indianapolis, 420 University Blvd, Indianapolis, IN 46202, USA; sadman.sazzad.md20@outlook.com; 4 Department of Endocrinology, Diabetes & Metabolism, Jacobs School of Medicine and Biomedical Sciences, The State University of New York, 705 Maple Road, Williamsville, NY 14221, USA; dr.shamimaakter@yahoo.com; 5 Department of Foundations of Medicine, NYU Grossman Long Island School of Medicine, 101 Mineola Blvd, Mineola, NY 11501, USA; sharif.smch@gmail.com; 6 Department of Applied Biosciences and Process Engineering, Anhalt University of Applied Sciences, Bernburger Strasse 55, 06366 Köthen, Germany; mja_shawon@yahoo.com; 7 Julius Bernstein Institute of Physiology, Medical School, Martin Luther University of Halle-Wittenberg, Magdeburger Str. 6, 06112 Halle, Germany

**Keywords:** citrus polyphenols, metabolic reprogramming, insulin resistance, mitochondrial dysfunction, endoplasmic reticulum stress, AMPK signaling, inflammatory cytokines, type 2 diabetes mellitus (T2DM)

## Abstract

Obesity-induced insulin resistance and type 2 diabetes mellitus (T2DM) represent complex systemic disorders marked by chronic inflammation, oxidative stress, mitochondrial dysfunction, and endoplasmic reticulum (ER) stress. These pathophysiological processes disrupt insulin signaling and β-cell function, leading to impaired glucose homeostasis across multiple organs. Conventional therapies often target isolated pathways, overlooking the intricate molecular crosstalk and organelle-level disturbances driving disease progression. Citrus-derived polyphenols—including hesperidin, naringenin, nobiletin, and tangeretin—have emerged as promising agents capable of orchestrating a multi-targeted “metabolic reprogramming.” These compounds modulate key signaling pathways, including AMPK, PI3K/Akt, NF-κB, and Nrf2, thereby enhancing insulin sensitivity, reducing pro-inflammatory cytokine expression, and restoring redox balance. Furthermore, they improve mitochondrial biogenesis, stabilize membrane potential, and alleviate ER stress by modulating the unfolded protein response (UPR), thus supporting cellular energy homeostasis and protein folding capacity. Evidence from preclinical studies and select clinical trials suggests that citrus polyphenols can significantly improve glycemic control, reduce oxidative and inflammatory markers, and preserve β-cell function. Their pleiotropic actions across molecular and organ-level targets position them as integrative metabolic modulators. This review presents a systems-level synthesis of how citrus polyphenols rewire metabolic signaling networks and organelle resilience, offering a holistic therapeutic strategy to mitigate the root causes of obesity-induced insulin resistance.

## 1. Introduction

It begins quietly, an expanding waistline, a subtle shift in blood sugar levels, a creeping fatigue. But behind these seemingly mundane signs brews a global biological crisis: the unstoppable rise in obesity-linked type 2 diabetes mellitus (T2DM). This twin epidemic, now termed “diabesity,” has grown into a full-blown metabolic pandemic [1,2]. As of 2023, more than 650 million adults are classified as obese, and over 500 million people live with diabetes, with projections pointing toward 700 million cases by 2045 [3,4,5,6]. Numbers, yes, but behind them lies a silent molecular war.

At the heart of this storm is a cascade of metabolic miscommunications. As adipose tissue expands, it not only stores excess energy but also morphs into a pro-inflammatory endocrine organ. Hypertrophied adipocytes release a torrent of cytokines such as TNF-α, IL-6, and MCP-1—recruiting macrophages and igniting a chronic inflammatory loop [7,8,9,10]. The result? The activation of NF-κB, JNK, and SOCS3, key molecular saboteurs that degrade insulin signaling, disrupt glucose uptake, and sow the seeds of insulin resistance across the liver, muscle, and pancreas [10,11,12,13]. But inflammation is only the beginning. With each calorie surplus, cells are flooded with free fatty acids, triggering lipotoxicity, ceramide accumulation, and a collapse in mitochondrial efficiency [14,15]. Overloaded mitochondria generate reactive oxygen species (ROS), feeding a cycle of oxidative damage that mutates proteins, disrupts redox balance, and disables insulin receptors [16,17]. Meanwhile, the endoplasmic reticulum (ER) tasked with protein folding buckles under metabolic pressure. UPR sensors, such as PERK, IRE1, and ATF6, are activated, prompting β-cells to undergo apoptosis and further exacerbating metabolic dysfunction in the body [18,19].

Despite our arsenal of pharmacological tools such as metformin, insulin, and GLP-1 agonists, there remains no cure for diabesity, only containment. These agents address symptoms but leave the root networks of dysfunction largely untouched [20]. This limitation has propelled research toward integrative, multi-targeted interventions derived from nature’s pharmacopeia.

Among these, citrus polyphenols have emerged as compelling candidates. In this review we introduce a unique framework—systemic metabolic reprogramming—which emphasizes how citrus polyphenols simultaneously recalibrate mitochondrial–ER crosstalk, immune–metabolic interactions, and organ-level adaptation [21]. These compounds work through AMPK, NF-κB, and Nrf2., and restore insulin sensitivity in the liver, adipose tissue, and skeletal muscle [21,22,23,24,25]. They modulate SIRT1–PGC1α pathways, stabilize mitochondrial membrane potential, and reduce ER stress through unfolded protein response signaling [21]. In doing so, they mitigate hyperglycemia, improve lipid metabolism, and prevent β-cell failure in preclinical models [25].

Previous reviews have largely focused on the antioxidant or single-pathway actions of citrus polyphenols. However, the concept of citrus flavonoids as systemic metabolic reprogrammers—simultaneously recalibrating mitochondrial–ER crosstalk, immune–metabolic signaling, and multi-organ adaptation—has not yet been synthesized. This review uniquely frames citrus polyphenols as network-level modulators within diabesity.

This review unpacks how citrus polyphenols intervene at key metabolic junctions, recalibrating molecular circuits, rejuvenating organelle function, and restoring systemic homeostasis. As our understanding deepens, these nature-derived compounds may offer more than dietary support; they may serve as network-based therapeutics in the fight against diabesity.

## 2. Pathophysiology of Obesity-Induced Diabetes

### 2.1. Lipotoxicity and Free Fatty Acid-Mediated Insulin Resistance

As the storm of chronic inflammation rages through expanding adipose tissue, another silent disruptor spreads through the bloodstream, free fatty acids (FFAs). In the obese state, basal lipolysis is persistently elevated, turning fat depots into active endocrine disruptors. These circulating FFAs are eagerly taken up by insulin-sensitive tissues, including the liver and skeletal muscle, where they undergo incomplete oxidation or are converted into lipotoxic intermediates such as diacylglycerols (DAGs) and ceramides [26,27].

These molecules act not merely as metabolic byproducts, but as intracellular saboteurs. By activating serine/threonine kinases such as c-Jun N-terminal kinase (JNK) and protein kinase C (PKC), they interfere with insulin signaling at its core, through the inhibitory serine phosphorylation of insulin receptor substrates (IRS1/2). The downstream result is twofold: in skeletal muscle, glucose uptake is impaired; in the liver, glucose production remains inappropriately elevated [28,29,30,31]. These defects form the biochemical bedrock of systemic insulin resistance. Yet, this mechanism is not universally expressed. A paradox remains: not all individuals with obesity develop T2DM. Beneath this variability lies a complex interplay of mitochondrial oxidative capacity, lipid partitioning, and intracellular buffering efficiency. Some individuals appear to be equipped with metabolic machinery capable of neutralizing lipotoxicity [32,33,34,35]. This variability suggests the urgent need for stratified approaches, where mitochondrial phenotype, not body mass index, guides therapeutic strategy.

In the liver, lipotoxicity takes on another form: hepatic insulin resistance. This defect impairs the suppression of gluconeogenic enzymes under insulin stimulation, resulting in fasting and postprandial hyperglycemia. Lipid-laden hepatocytes, struggling under the weight of FFA influx, experience impaired AKT activation and PKC-mediated insulin receptor dysfunction [36,37,38]. Meanwhile, Kupffer cells, the liver’s resident macrophages, release inflammatory cytokines that compound the damage and amplify insulin resistance [39]. Still, one critical question lingers: is hepatic steatosis the cause or merely a companion of insulin resistance? Although these conditions often coexist, emerging evidence suggests that hepatic inflammation and lipotoxicity, not simple steatosis, are the more potent drivers of metabolic impairment [40,41,42]. As such, targeting inflammatory and lipid-signaling pathways within the liver may offer greater therapeutic promise than attempts to merely de-fat the organ.

### 2.2. Oxidative Stress, Mitochondrial Dysfunction, and ER Disruption

As lipotoxic intermediates sabotage insulin signaling from the surface, a deeper disturbance brews within the cell—oxidative stress. In obesity, the ectopic deposition of lipids in liver and skeletal muscle overburdens cellular respiration, particularly within the mitochondria. The result is an overproduction of reactive oxygen species (ROS), driven not only by mitochondrial electron transport chain leakage but also by upregulated NADPH oxidase (NOX) activity [43,44,45] [Table 1]. 

These ROS act as rogue messengers, damaging proteins, lipids, and DNA, and activating redox-sensitive inflammatory pathways, such as NF-κB and JNK, which further disrupt insulin receptor signaling [74,75]. Moreover, elevated oxidative stress impairs mitochondrial ATP production, undermines metabolic flexibility, and sets the stage for mitochondrial dysfunction—a central node in the pathogenesis of insulin resistance [76,77]. Yet, ROS are not universally destructive. At physiological levels, they function as essential signaling molecules, fine-tuning insulin sensitivity and energy flux. It is only when their production surpasses the buffering capacity of antioxidant systems that they become pathological. This duality makes ROS modulation a therapeutic tightrope—too much suppression may blunt necessary signaling, while too little invites metabolic collapse [77,78].

But the damage does not end at the mitochondria. Just beyond lies another vulnerable organelle, the endoplasmic reticulum (ER), which is tasked with protein folding, calcium storage, and lipid biosynthesis. In obesity, ER homeostasis is frequently disrupted, as excess nutrients and oxidative signals trigger the unfolded protein response (UPR). Key sensors, such as PERK, IRE1, and ATF6, become chronically activated, leading to maladaptive signaling, impaired insulin receptor trafficking, and even β-cell apoptosis [78,79,80].

Compounding the crisis, ER stress and mitochondrial dysfunction do not act in isolation. Instead, they engage in bidirectional cross-talk, where ROS produced by mitochondrial overload exacerbate ER stress, and ER stress in turn destabilizes mitochondrial dynamics and calcium homeostasis [81,82]. This self-reinforcing loop amplifies cellular distress, propelling the insulin-resistant phenotype forward.

Thus, in the obese insulin-resistant state, the combined dysfunction of mitochondria and ER forms a molecular sinkhole, collapsing the delicate architecture of intracellular communication and energy balance. Targeting this axis, mitochondrial redox tuning, and restoration of ER homeostasis may be essential for effective metabolic reprogramming.

### 2.3. β-Cell Compensation and Failure

As insulin resistance deepens in peripheral tissues, the metabolic burden shifts to the pancreas. For a time, the β-cells rise to the occasion. In early obesity, these endocrine sentinels respond to rising glycemic demand with compensatory hypersecretion of insulin, working overtime to preserve normoglycemia [83,84].

But this heroism comes at a cost. With chronic exposure to hyperglycemia and elevated FFAs, a biochemical storm known as glucolipotoxicity, β-cells begin to falter. Initially, their function deteriorates: insulin granule synthesis declines, secretory machinery loses fidelity, and glucose-stimulated insulin secretion becomes blunted [85,86].

Over time, this dysfunction becomes irreversible. A convergence of molecular insults, oxidative stress, ER stress, amyloid deposition, and islet-localized inflammation drives β-cells toward apoptosis and loss of mass [87]. Mitochondrial inefficiency further impairs insulin granule exocytosis, while pro-inflammatory cytokines such as IL-1β and IFN-γ infiltrate islets and exacerbate cell death [88]. Eventually, the tipping point is reached: the remaining β-cell pool is no longer sufficient to compensate. Glucose levels rise unchecked, and what began as a compensatory adaptation gives way to overt T2DM [89]. Yet the fate of the β-cell may not be entirely sealed. Some dysfunction appears reversible, at least in early stages, raising hope for therapeutic intervention. GLP-1 receptor agonists, for example, have been shown to enhance β-cell function, promote insulin gene expression, and even reduce apoptosis. However, restoring β-cell mass, not just improving function, remains a challenging task [90,91].

To this end, regenerative strategies such as β-cell neogenesis, trans-differentiation, and reprogramming of other pancreatic cells are under active exploration. While preclinical data offer tantalizing glimpses of success, translation into durable human therapies is still in its infancy [92]. Thus, the story of the β-cell in diabesity is one of early heroism, gradual unraveling, and an uncertain future. Preserving its function and perhaps, one day, restoring its mass may hold the key to altering the trajectory of metabolic disease.

### 2.4. Adipose Tissue Dysfunction and Immune Activation

As systemic insulin resistance develops, one of its earliest instigators lies buried in the expansion of fat depots. In the setting of chronic overnutrition, adipocytes enlarge through hypertrophy, outpacing their oxygen supply and creating localized hypoxia. This cellular stress activates hypoxia-inducible factors (HIFs) and initiates an inflammatory cascade [93,94].

**Figure 1:**
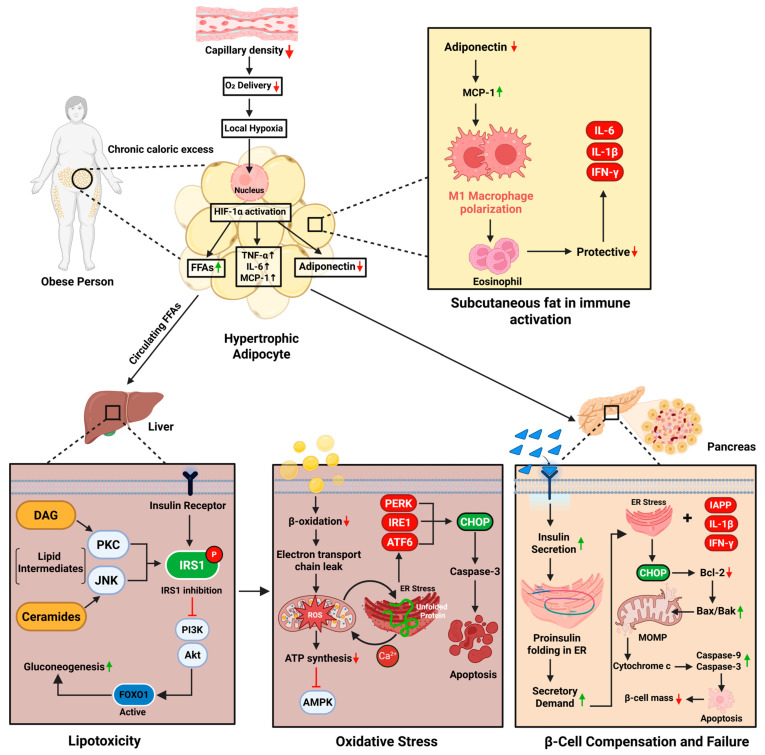
Schematic representation of the mechanisms linking obesity to β-cell failure and T2DM. Chronic caloric excess induces adipocyte hypertrophy and local hypoxia, activating HIF-1α and promoting the release of FFAs, TNF-α, IL-6, and MCP-1, while reducing adiponectin. This triggers M1 macrophage polarization and inflammation in adipose tissue. Circulating FFAs and lipid intermediates reach the liver, activating PKC/JNK pathways and impairing IRS1–PI3K–Akt signaling, resulting in increased gluconeogenesis. Concurrently, impaired β-oxidation and mitochondrial dysfunction lead to ROS accumulation, ER stress, and apoptosis. In the pancreas, inflammatory and ER stress signals impair insulin synthesis and activate mitochondrial apoptotic pathways, reducing β-cell mass and insulin secretion—contributing to hyperglycemia and T2DM. (Image created with biorender.com).

Stressed adipocytes begin secreting danger signals, including chemokines such as monocyte chemoattractant protein-1 (MCP-1), which draw immune cells into the adipose microenvironment. There, monocytes differentiate into classically activated (M1) macrophages, forming crown-like structures around dying adipocytes and releasing a storm of pro-inflammatory cytokines: TNF-α, IL-6, and IL-1β [93]. These cytokines interfere with insulin receptor signaling by inhibiting the serine phosphorylation of IRS1, ultimately contributing to systemic insulin resistance [95]. At the same time, levels of adiponectin decline—an insulin-sensitizing adipokine with potent anti-inflammatory and vascular-protective properties. This drop further tips the balance toward a pro-inflammatory adipose phenotype, perpetuating metabolic dysfunction [96] [Figure 1].

But this immune–metabolic dialog is far from uniform. Depot-specific differences—between visceral, subcutaneous, and brown adipose tissue—shape the inflammatory landscape, as do the plastic and dynamic phenotypes of resident immune cells. In some depots, regulatory T cells, eosinophils, and alternatively activated (M2) macrophages attempt to counterbalance inflammation, though their roles remain incompletely understood [93]. As this immunological remodeling unfolds, translational researchers face a challenge: current animal models and cell lines oversimplify the dynamic complexity of human adipose tissue. The precise orchestration of immune-adipocyte interactions during obesity progression and weight loss remains a frontier of investigation, requiring refined model systems and longitudinal tissue profiling [97,98,99].

Thus, the immune–adipocyte axis stands as a central pillar of metabolic inflammation, a battlefield where energy sensing, immune signaling, and hormonal control collide.

## 3. Citrus Polyphenols in Metabolic Reprogramming

### 3.1. Sour Yet Sweet Salvation: How Citrus Polyphenols Rewire Diabetic Metabolism

Until now, the story has unfolded as a progressive unraveling of metabolic order, characterized by lipotoxicity, oxidative stress, mitochondrial dysfunction, inflammatory infiltration, and β-cell failure [15,100,101]. But nature, too, offers its counterstrike. Quietly nestled among citrus fruits, a class of polyphenolic compounds has shown a surprising capacity to engage the metabolic network, not as blunt tools, but as precise modulators capable of reprogramming disordered pathways [21].

**Figure 2:**
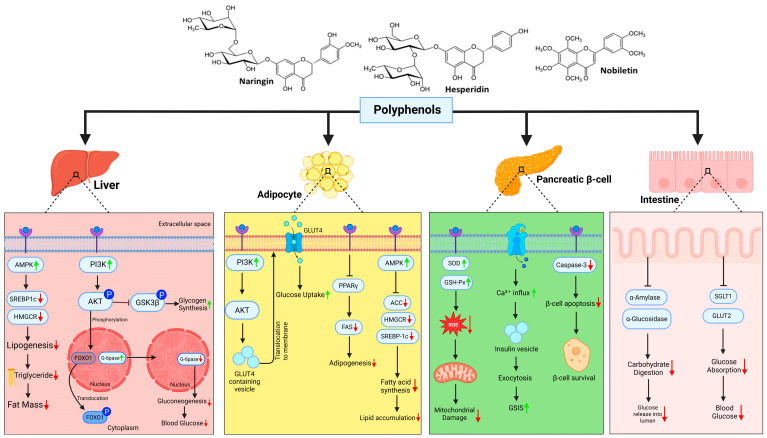
Multi-organ effects of citrus fruit polyphenols in obesity-induced diabetes. In the liver, polyphenols activate AMPK and PI3K/AKT signaling, suppress SREBP1c and HMGCR, reduce lipogenesis, and inhibit gluconeogenic enzymes (G6Pase, FOXO1), thereby lowering hepatic glucose output. In adipocytes, they enhance PI3K–AKT–GLUT4 translocation to improve glucose uptake and downregulate PPARγ, FAS, and SREBP1c to reduce adipogenesis and lipid accumulation. In pancreatic β-cells, polyphenols upregulate antioxidant enzymes (SOD, GSH-Px), reduce ROS, inhibit caspase-3, and promote Ca^2+^ influx, enhancing insulin secretion and survival. In the intestine, they inhibit α-amylase and α-glucosidase, slowing carbohydrate digestion and reducing postprandial glycemia via SGLT1/GLUT2 downregulation. (Image created with biorender.com).

In addition to functional outcomes, the structural features of citrus polyphenols are critical in explaining their biological activity. Figure 2 presents the chemical structures of the major flavonoids discussed in this review, including hesperidin, naringenin, nobiletin, tangeretin, eriocitrin, quercetin, and neohesperidin. These compounds share a flavanone or flavone backbone, with variation in hydroxylation, glycosylation, and methoxylation patterns. Such structural differences underlie their capacity to modulate redox balance and metabolic pathways.

Hesperidin, a glycosylated flavanone, possesses hydroxyl groups at the 5- and 7-positions of the A-ring and initiates its metabolic rescue by activating AMPK the cell’s energy sensor. This activation restores PI3K/Akt signaling, enabling the translocation of GLUT4 in skeletal muscle and adipose tissue, thereby reviving insulin responsiveness [21,102,103,104]. Simultaneously, hesperidin attenuates ROS accumulation and boosts endogenous antioxidants like SOD, GPx, and catalase, relieving oxidative stress and restoring mitochondrial integrity key to breaking the self-perpetuating cycle of insulin resistance [103]. Quercetin, abundant in citrus and other plants, reinforces this network-level modulation. It not only improves glycemic control via PI3K/Akt and IRS1 restoration, but also blocks NF-κB-mediated inflammation, decreasing expression of TNF-α and IL-6—two upstream drivers of adipose and hepatic insulin resistance [104,105]. Quercetin and eriocitrin, with multiple catechol groups, provide potent metal-chelating and antioxidant properties. These structural insights highlight common factors—redox modulation, lipid solubility, and receptor interactions—that enable citrus flavonoids to reverse metabolic dysfunction [106,107].

Then comes naringenin, the aglycone of naringin, which enters the liver and shifts metabolic flux toward fatty acid oxidation. It activates PPARα, downregulates SREBP-1c, and inhibits JNK signaling, thereby reducing lipogenesis, inflammation, and insulin resistance in hepatocytes [21,108,109]. In skeletal muscle, it reinforces AMPK signaling, improving glucose uptake and restoring oxidative capacity [66,110].

Tangeretin, sourced from citrus peels, contributes by modulating adipokine profiles—notably restoring adiponectin levels, which enhances insulin sensitivity through both AMPK activation and Pparγ modulation [111,112]. It also suppresses oxidative stress markers in insulin-sensitive tissues, reinforcing mitochondrial health and preserving metabolic signaling fidelity [113]. Kaempferol, while less abundant, wields a unique role in suppressing hepatic gluconeogenesis. By activating AMPK, it downregulates PEPCK and G6Pase, two key enzymes responsible for excess hepatic glucose output in insulin-resistant states [114,115,116,117]. Through this mechanism, it reprograms hepatic energy flow and corrects fasting hyperglycemia [116,117]. Rutin, structurally similar to quercetin, amplifies this ensemble response by restoring mitochondrial biogenesis, enhancing SIRT1–PGC1α signaling, and mitigating oxidative stress, particularly in the pancreas and liver [116].

Together, these compounds do not simply oppose insulin resistance—they rewire the system. Through synergistic targeting of AMPK, PI3K/Akt, NF-κB, PPARs, SIRT1, and UPR components, citrus polyphenols restore cellular energy sensing, reduce inflammatory signaling, and regenerate insulin sensitivity across the liver, muscle, adipose, and pancreas tissues. Their strength lies not in singular action, but in their ability to act in concert, modulating interlinked metabolic pathways at the core of diabesity. Thus, citrus polyphenols represent more than nutraceuticals. They are biochemical architects, reshaping the very systems that obesity and hypernutrition seek to dismantle.

### 3.2. Citrus Polyphenols and Inflammatory Reprogramming in Diabesity

In the evolving landscape of diabesity, inflammation emerges not as a byproduct but as a driving force—a molecular amplifier that sustains insulin resistance and disrupts metabolic homeostasis. The epicenter of this inflammatory storm lies within adipose tissue, where immune–metabolic crosstalk distorts insulin signaling at every level.

TNF-α, first identified as a pivotal factor in obese adipose tissue by Hotamisligil et al. [118], sets this cascade in motion [119,120]. As previously described in Section 3.1, citrus polyphenols suppress NF-κB nuclear translocation and normalize IRS1–Akt signaling [121,122,123]. Simultaneously, NF-κB, a master inflammatory transcription factor, translocates to the nucleus, driving the expression of cytokines such as IL-6, MCP-1, and iNOS, which reinforce metabolic dysfunction and immune infiltration [7,124,125]. Amid this inflammatory gridlock, citrus polyphenols have emerged not merely as suppressors of inflammation but as metabolic circuit breakers, capable of disconnecting inflammatory feedback loops and restoring intracellular communication [21].

Naringin, hesperidin, and quercetin have all been shown to suppress NF-κB nuclear translocation, thereby silencing pro-inflammatory transcriptional programs at their source. In murine and cellular models, these polyphenols reduce the expression of COX-2, iNOS, and inflammatory cytokines, thereby reshaping the cytokine environment from a pro-inflammatory to a homeostatic one [126,127]. Further upstream, they interfere with the AGE-RAGE signaling pathway, a critical amplifier of chronic inflammation and oxidative stress in metabolic tissues. Naringin and hesperidin downregulate RAGE expression and reduce AGE accumulation, blunting ROS generation and downstream cytokine bursts [128,129,130]. Importantly, these molecular insights are not confined to the bench. Clinical data reveal that citrus bioflavonoid complexes significantly lower plasma levels of IL-6 and TNF-α, as well as biomarkers of DNA oxidative damage, such as 8-OHdG, suggesting a systemic reprogramming of inflammatory tone in human subjects [131]. This anti-inflammatory reprogramming does not merely reduce cytokines; it restores the integrity of insulin signaling. With NF-κB silenced and JNK/IKKβ activity reduced, IRS1 phosphorylation normalizes, Akt activation is restored, and glucose uptake resumes. This is metabolic recalibration—not via single-point intervention, but through network-level remodeling of the immune–metabolic interface.

Thus, citrus polyphenols act as molecular disruptors of inflammatory inertia, targeting not only the messengers of inflammation but the architecture that sustains it. Their ability to realign immune and metabolic signaling pathways elevates them from anti-inflammatory agents to true agents of metabolic reprogramming.

### 3.3. Role of Citrus in Mitochondrial Health and Endoplasmic Reticulum Stress: Restoring Protein Homeostasis

In the battle against metabolic disease, restoring energy balance and cellular homeostasis is no longer just a downstream goal; it is a frontline strategy. As insulin signaling falters and inflammation mounts, the cell’s two central organelles for metabolic coordination—the mitochondria and the endoplasmic reticulum (ER) emerge as critical nodes of dysfunction [21]. Their deterioration underlies the systemic collapse in energy sensing, protein folding, and glucose regulation seen in obesity-induced T2DM.

At the heart of this collapse lies mitochondrial dysfunction, driven by nutrient overload, lipotoxicity, and oxidative stress. In metabolically active tissues—especially liver, adipose, and muscle—excess free fatty acids impair mitochondrial respiration, increase reactive oxygen species (ROS), and destabilize membrane potential, culminating in reduced ATP synthesis and suppression of insulin-stimulated glucose uptake [132,133].

Here, citrus polyphenols—particularly naringenin, hesperidin, and tangeretin—intervene as metabolic stabilizers. Their actions go beyond antioxidant defense. By activating the Nrf2–ARE pathway, they enhance endogenous antioxidant enzyme expression (SOD, catalase, GPx) and limit mitochondrial ROS accumulation [134,135]. More importantly, these polyphenols stimulate PGC-1α, NRF1, and TFAM, transcriptional regulators of mitochondrial biogenesis and energy renewal, reprogramming the cell toward oxidative resilience [134].

Naringenin, in particular, has been shown to preserve mitochondrial membrane potential, boost ATP output, and regulate PINK1/Parkin-mediated mitophagy, thereby eliminating dysfunctional mitochondria and sustaining metabolic flux in hepatocytes and myocytes under lipotoxic conditions [136,137,138]. Meanwhile, hesperidin enhances mitochondrial efficiency and prevents apoptotic signaling by reducing cytochrome c release and caspase activation, thereby preserving cell viability in the face of metabolic overload [139].

Yet mitochondria are not isolated actors. Their function is tightly intertwined with that of the endoplasmic reticulum (ER)—an organelle responsible for protein folding, lipid biosynthesis, and calcium signaling [140]. Under chronic nutrient stress, the ER accumulates misfolded proteins, triggering unfolded protein response (UPR) pathways. If unresolved, this stress spills over into apoptosis, fueling insulin resistance and β-cell failure [99,141,142,143].

Once again, citrus flavonoids offer a path to restoration. Compounds like naringenin and hesperidin selectively attenuate the PERK–eIF2α, IRE1–XBP1, and ATF6 arms of the UPR, preventing the maladaptive amplification of ER stress. In high-fat-diet models, naringenin reduces CHOP expression, a pro-apoptotic UPR marker, and restores adaptive ER signaling, thereby rescuing β-cell and hepatocyte function [141,144,145].

These polyphenols also enhance GRP78/BiP expression, boosting chaperone capacity and facilitating correct protein folding under stress conditions [140,146]. Additionally, by modulating ER-mitochondrial crosstalk, citrus compounds support coordinated calcium transfer, optimize oxidative metabolism, and prevent apoptosis—a level of control that aligns precisely with systemic metabolic reprogramming [147,148,149].

Thus, citrus polyphenols do not merely suppress damage; they restore the intracellular architecture upon which metabolic homeostasis depends. By recalibrating mitochondrial bioenergetics, enhancing mitophagy, stabilizing ER folding capacity, and reinforcing cross-organelle signaling, they initiate a cellular response that rewires the failing metabolic circuitry of diabesity from within.

### 3.4. Free Radicals, Oxidative Stress, and Citrus Polyphenols: A Natural Line of Defense

As the metabolic storm deepens, a new biochemical disruptor takes center stage: oxidative stress. While reactive oxygen species (ROS) serve as transient messengers under physiological conditions, chronic metabolic overload transforms them into agents of cellular sabotage, eroding the signaling pathways that maintain metabolic equilibrium.

**Figure 3:**
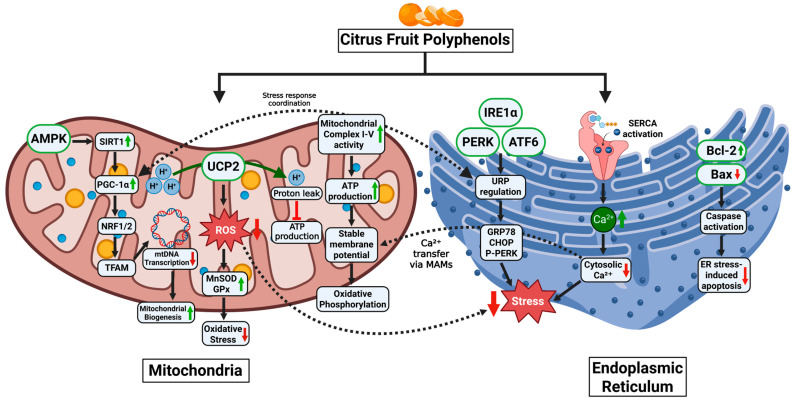
Citrus polyphenols regulate mitochondrial and endoplasmic reticulum (er) homeostasis under metabolic stress. This diagram illustrates the coordinated protective effects of citrus fruit polyphenols on mitochondrial and ER function. In mitochondria, polyphenols activate the AMPK–SIRT1–PGC-1α pathway, enhancing mitochondrial biogenesis via NRF1/2 and TFAM, and reducing ROS through upregulation of MnSOD and GPx. They stabilize the mitochondrial membrane potential, support ATP production, and improve complex I/IV activity while reducing proton leak and oxidative stress. In the ER, citrus polyphenols alleviate ER stress by modulating UPR path ways (PERK, IRE1α, ATF6), reducing expression of stress markers (CHOP, p-PERK), enhancing SERCA-mediated Ca^2+^ influx, and promoting ER–mitochondria Ca^2+^ transfer. They also inhibit apoptosis by increasing Bcl-2 and suppressing Bax and caspase activation. Together, these actions maintain cellular homeostasis and prevent metabolic dysfunction. (Image created with biorender.com).

In obesity-induced diabetes, the overproduction of ROS—particularly superoxide (O_2_^−^), hydrogen peroxide (H_2_O_2_), and hydroxyl radicals (•OH)—disrupts insulin receptor integrity, impairs glucose uptake, and accelerates β-cell dysfunction [45,150,151]. These free radicals are not isolated threats; they are embedded within a vicious cycle where lipotoxicity, inflammation, and mitochondrial inefficiency reinforce oxidative damage, amplifying insulin resistance across tissues [152] [Figure 3]. 

The collapse of redox homeostasis is especially destructive in insulin-sensitive cells like hepatocytes, myocytes, and adipocytes, where antioxidant defense systems are often overwhelmed [67,153]. The antioxidant enzymatic triad—superoxide dismutase (SOD), catalase (CAT), and glutathione peroxidase (GPx)—is essential for neutralizing ROS. However, in metabolic disease, these systems are either underexpressed or dysfunctional, allowing oxidative stress to accumulate unchecked [150,151].

At this critical juncture, citrus polyphenols emerge as a dual-action metabolic defense—scavengers of free radicals and regulators of redox-responsive gene expression. Unlike synthetic antioxidants that act in isolation, these natural compounds integrate into the metabolic network, restoring antioxidant capacity from within [154,155]. For example, supplementation with Citrus unshiu peel extract has been shown to significantly increase hepatic levels of SOD, CAT, GPx, and glutathione (GSH) in high-fat-diet-fed mice, leading to reductions in lipid peroxidation and improvement in insulin sensitivity [156]. This restoration of redox balance helps normalize insulin signaling cascades and suppress stress-induced kinase activation, such as JNK and IKKβ, which are known inhibitors of insulin receptor substrates [154,157].

The effect is not compound-specific but rather synergistic. Flavonoids such as naringenin, hesperidin, and quercetin, abundant in citrus species like Citrus paradisi, Citrus sinensis, and Citrus maxima, not only scavenge ROS directly but also activate the Nrf2–ARE pathway—a master regulator of endogenous antioxidant gene expression [53,155,158]. Activation of Nrf2 leads to increased transcription of SOD, GPx, and CAT, reinforcing intracellular defenses at the transcriptional level [158].

**Figure 4:**
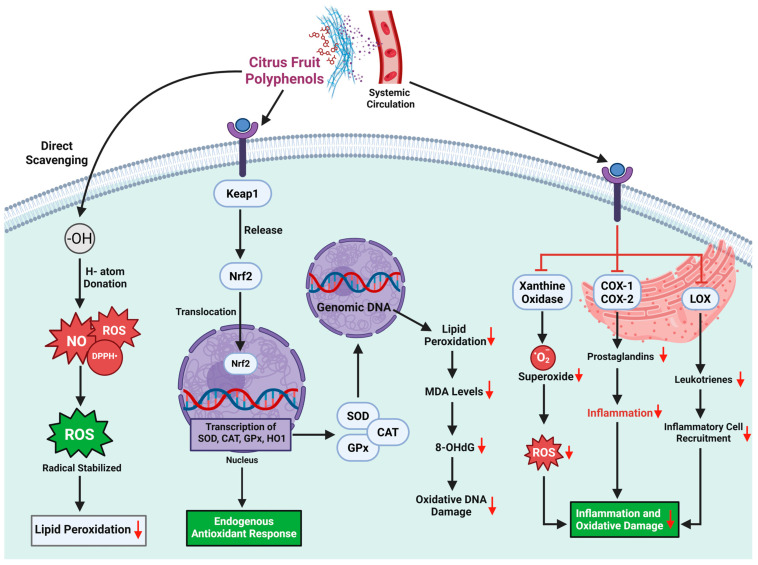
Citrus fruit polyphenols mitigate oxidative stress in the liver. This schematic depicts how citrus polyphenols exert antioxidant and anti-inflammatory effects to protect hepatic tissue from oxidative damage. On the left, citrus polyphenols directly scavenge reactive oxygen species (ROS), DPPH^+^ (2,2-diphenyl-1-picrylhydrazyl), and NO radicals via hydrogen atom donation, preventing lipid peroxidation and radical propagation. Simultaneously, they activate the Keap1–Nrf2 pathway, promoting Nrf2 release and nuclear translocation. Nrf2 then binds to antioxidant response elements (ARE), upregulating endogenous antioxidants such as SOD, CAT, GPx, and HO-1. This enhances hepatic ROS detoxification and reduces oxidative stress markers like malondialdehyde (MDA) and 8-hydroxy-2′-deoxyguanosine (8-OHdG). On the right, citrus polyphenols inhibit xanthine oxidase as well as COX-1/COX-2 and LOX enzymes, decreasing prostaglandin and leukotriene synthesis and limiting inflammatory cell infiltration. Together, these mechanisms mitigate oxidative DNA damage and inflammation, preventing chronic liver injury. (Image created with biorender.com).

Notably, extracts of red pummelo (Citrus maxima) demonstrated 20–40% higher free radical scavenging capacity than vitamin C or synthetic antioxidants like BHA, underlining the superior efficacy of these phytochemical complexes in redox modulation [159]. Thus, oxidative stress is not merely a consequence of metabolic dysfunction; it is a pathogenic amplifier of insulin resistance and systemic energy imbalance. By disrupting this oxidative-inflammation loop, citrus polyphenols do more than clean up molecular debris; they restore the redox environment required for metabolic precision. In doing so, they enable cells to re-establish insulin responsiveness, protect β-cell integrity, and suppress the molecular triggers of inflammation. This metabolic recalibration through antioxidant programming firmly positions citrus-derived polyphenols as frontline defenders in the systems-level war against diabesity (Figure 4).

### 3.5. Translating Mechanisms to Humans: Clinical Evidence of Citrus Polyphenol-Driven Metabolic Reprogramming

Having followed citrus polyphenols through cellular landscapes—where they extinguish oxidative stress, rewire mitochondrial signaling, restore protein-folding homeostasis, and silence inflammatory circuits—we arrive at the final test: do these molecular shifts translate to human physiology?

Clinical trials provide preliminary but encouraging signals that citrus polyphenols may recalibrate metabolic function. However, most studies remain limited by small sample sizes, short durations, and population heterogeneity. Therefore, current evidence should be regarded as exploratory, highlighting the need for multicenter RCTs and large longitudinal studies to establish efficacy [58,159] [Table 2]. 

In a double-blind, randomized clinical trial, 500 mg/day of hesperidin administered over three weeks significantly reduced TNF-α and VCAM-1, thereby restoring endothelial function—a proxy for systemic inflammatory tone and insulin sensitivity [22]. Importantly, these effects are not isolated to the vasculature but echo upstream changes in NF-κB signaling and insulin receptor substrate activity, consistent with earlier mechanistic studies.

While current clinical studies remain limited in scale and duration, ongoing large-scale randomized controlled trials are investigating citrus flavonoid supplementation in populations with metabolic syndrome and NAFLD. These trials are expected to provide more definitive evidence on efficacy, dose–response relationships, and long-term safety. Such future research will be essential for translating the promising mechanistic and preclinical findings into clinical practice.

Naringin, too, has moved from bench to bedside. Oral supplementation in patients with metabolic syndrome resulted in reductions in total cholesterol, LDL-C, and triglycerides, while increasing HDL-C, an indirect yet essential component of insulin signaling restoration [160,161]. These lipid profile improvements align with its known PPARγ activation and inhibition of JNK signaling, bridging preclinical mechanisms with clinical outcome [25,162].

In a dietary intervention study, individuals with T2DM who followed a Mediterranean diet enriched with citrus fruits experienced a reduction in HbA1c from 7.1% to 6.8% over 12 weeks, indicating an improvement in long-term glycemic control through enhanced insulin sensitivity and restored glucose uptake mechanisms [163].

Perhaps most compelling is the clinical validation of bergamot polyphenolic fraction (BPF)—a mixture rich in neoeriocitrin, naringin, and neohesperidin. In hyperlipidemic and prediabetic subjects, BPF not only lowered LDL-C and improved glucose metabolism, but achieved these results with efficacy comparable to statin therapy, yet without pharmacological side effects [164,165].

These studies converge on a single, powerful theme: citrus polyphenols act at the systems level, engaging molecular targets already mapped in preclinical models—AMPK, NF-κB, PI3K/Akt, PPARs—to bring about clinically meaningful metabolic restoration. Their ability to reduce inflammatory biomarkers, improve insulin action, recalibrate lipid metabolism, and preserve vascular function confirms their role not just as dietary supplements, but as translational agents in the therapeutic modulation of diabesity. And so, from the molecular to the metabolic, from mitochondria to macrosystems, citrus polyphenols close the loop: a natural, multifaceted, clinically validated reprogramming of the diseased metabolic network.

**Table 2:** Clinical studies of the effect of citrus polyphenols on metabolic reprogramming. This table summarizes randomized clinical trials, dietary interventions, or meta-analyses evaluating hesperidin, naringin, neoeriocitrin, and other citrus-derived polyphenols. Reported outcomes include changes in lipid profile, glycemic markers, inflammatory cytokines, liver fat, and endothelial function. Study size, intervention dose, and duration are also included.

Compound(s)	Study Design and Population	Intervention Details (Dose and Duration)	Reported Outcomes	Mechanism of Action	Source
Neoeriocitrin, Naringin, Neohesperidin	RCT, overweight adults with MASLD (*n* = 80)	Bergamot extract (500–1000 mg/day, 12 weeks)	↓ Liver fat content (−18%), ↓ body weight (−5% vs. placebo)	Enhances bile flow; antioxidant activity reduces oxidative stress	[166]
Hesperidin, Naringin, Neohesperidin	RCT, metabolic syndrome (*n* = 95)	Mixed citrus extracts (500 mg/day, 8 weeks)	↑ Endothelial function (FMD ↑12%), improved vascular tone	Antioxidant effects improve vascular inflammation and nitric oxide availability	[167]
Hesperidin → Hesperetin; SCFAs	Clinical trial, healthy volunteers (*n* = 40)	Citrus fruit extract (500 mg/day, 4 weeks)	Gut microbiota modulation: ↑ Bifidobacterium, ↑ SCFA production; ↓ systemic inflammation	Hesperidin metabolized to hesperetin → SCFA-mediated endothelial protection and anti-inflammatory response	[168]
Flavones, Flavanones, Oleuropein	RCT, high-CV-risk adults (*n* = 120)	Citrus + olive polyphenol mix (500 mg/day, 12 weeks)	↓ Cardiovascular risk biomarkers; ↓ hs-CRP (−20%); improved metabolic-inflammatory profile	Antioxidant activity; NF-κB inhibition	[169]
Hesperidin, Naringin, Oleuropein	RCT, adults with dyslipidemia (*n* = 72)	Citrus + olive leaf extracts (500 mg/day, 10 weeks)	↓ LDL oxidation (−12%), ↓ TNF-α (−18%), ↓ IL-6 (−15%)	Free radical scavenging; cytokine modulation	[170]
Hesperidin	RCT, obese adults (*n* = 64)	Orange juice (Citrus sinensis, ~500 mL/day, 12 weeks)	↓ BMI (−1.2 kg/m^2^), ↓ waist circumference (−3.4 cm), ↓ IL-1β, IL-6, TNF-α	Inhibits pro-inflammatory cytokine release; antioxidant endothelial protection	[171]
Hesperidin	RCT, NAFLD patients (*n* = 82)	Hesperidin 1 g/day + lifestyle changes (12 weeks)	↓ Liver fat (−22%), ↓ ALT (−30%), ↓ TG (−18%), ↓ weight (−4 kg)	NF-κB inhibition; ↓ TNF-α, ↓ hs-CRP	[172]
Hesperidin (meta-analysis)	Meta-analysis of RCTs (*n* = 525 metabolic subjects)	Hesperidin (500–1000 mg/day, 4–12 weeks)	↓ TG, ↓ TC, ↓ LDL (especially in BMI >30); ↓ TNF-α, ↓ IL-6 at higher doses	Anti-inflammatory; lipid-lowering	[173]
Orange juice (flavonoids)	4-week RCT, MASLD patients (*n* = 62)	Orange juice (500 mL/day)	↓ Liver steatosis (by FibroScan), ↓ GGT (−10%)	Antioxidant effect; modest anti-inflammatory	[174]
Flavonoid-enriched orange juice	RCT, metabolic syndrome patients (*n* = 48)	Enriched OJ (500 mL/day, 6 weeks)	↑ Antioxidant status (TAC ↑15%), improved glycemic trend	↓ CRP, ↓ endothelial inflammation	[175]
Hesperidin	RCT, vascular function study (*n* = 24 metabolic syndrome patients)	Hesperidin 500 mg/day, 3 weeks	↑ FMD (+12%), ↓ IL-6 (−15%), ↓ TNF-α (−12%)	↑ NO bioavailability; ↓ inflammatory cytokines	[22]
Eriomin^®^ (Eriocitrin)	Crossover RCT, prediabetes patients (*n* = 103)	Eriomin^®^ 200–500 mg/day, 12 weeks	↓ FBG (−5.5 mg/dL), ↓ HOMA-IR (−18%), ↑ GLP-1 (+15%), ↑ adiponectin (+20%)	↓ IL-6, TNF-α, hs-CRP	[176]
Polyphenols incl. Naringenin	Meta-analysis in NAFLD patients (12 RCTs, *n* ≈ 950)	Various flavonoids (6–12 weeks)	↓ BMI, ↓ ALT (−12%), ↓ AST (−10%), ↓ TG (−18%), ↓ TNF-α (−14%)	Anti-inflammatory; metabolic reprogramming	[177]

## 4. Role of Lipoproteins in Diabetes and the Impact of Citrus Polyphenols

Lipoproteins play a central role in the pathophysiology of insulin resistance and type 2 diabetes [178]. In diabetic states, apoB-containing lipoproteins such as very-low-density lipoprotein (VLDL) and low-density lipoprotein (LDL) are elevated, driving lipid overload in the liver and peripheral tissues. These particles are highly enriched in triglyceride-derived lipid hydroperoxides, which amplify oxidative stress and vascular inflammation [179]. In contrast, levels of high-density lipoprotein (HDL) are reduced, and HDL function is often impaired. Dysfunctional HDL loses its antioxidant and anti-inflammatory properties, limiting its capacity to neutralize lipid peroxides and further exacerbating the pro-inflammatory state [180].

Citrus polyphenols have been reported to beneficially modulate lipoprotein metabolism. Hesperidin supplementation in humans reduces plasma LDL and triglycerides while modestly increasing HDL levels [23]. Bergamot polyphenolic fraction (rich in neoeriocitrin, naringin, and neohesperidin) lowers apoB-containing lipoproteins and improves HDL function [181]. Animal studies demonstrate that naringin and nobiletin suppress hepatic VLDL secretion and enhance reverse cholesterol transport. These actions collectively reduce circulating lipid hydroperoxides and restore lipoprotein redox balance [182].

By targeting both lipoprotein levels and lipoprotein functionality, citrus polyphenols address an under-recognized contributor to metabolic dysfunction in diabetes [183]. Their ability to restore HDL’s antioxidant capacity and attenuate VLDL-derived oxidative stress provides a mechanistic link between polyphenol chemistry, lipid metabolism, and vascular protection. This integration expands the translational relevance of citrus polyphenols beyond glucose homeostasis to the broader spectrum of cardiometabolic risk.

## 5. Conclusions and Future Direction

In the orchestra of cellular life, metabolic reprogramming is the conductor’s baton, guiding the tempo of nutrient sensing, mitochondrial energy flow, insulin action, and inflammatory resolution. But in obesity-induced diabetes, the rhythm descends into disarray. The once-synchronized network of metabolic signals becomes a cacophony of oxidative stress, unfolded proteins, inflammatory feedback, and lipotoxic derailment.

Yet, amid this dysfunction, a new score emerges, one not composed in laboratories but grown in groves of citrus, where nature has refined its symphony of healing.

Throughout this review, we followed citrus-derived polyphenols as they stepped into this metabolic dissonance, not as singular antioxidants or hypoglycemic agents, but as systemic modulators orchestrating a return to cellular harmony. From naringenin’s mitochondrial recalibration to hesperidin’s anti-inflammatory tuning, these flavonoids reactivated dormant pathways, rewired dysfunctional circuits, and restored homeostatic balance at every biological level—liver, adipose, muscle, pancreas, and beyond.

This review integrates evidence across molecular, organelle, and clinical dimensions to present citrus polyphenols as systemic reprogramming agents. This framework underscores their role not merely as antioxidants but as network-based therapeutic candidates.

Their strength lies not in pharmacological force, but in biological fluency. Citrus polyphenols do not attack the system—they speak its language, modulating master nodes like AMPK, PPARγ, Nrf2, and NF-κB, enabling tissues to heal from within. This systems-level approach is what places them at the forefront of a new therapeutic paradigm: one where nutritional networks, not isolated molecules, reshape disease trajectories.

Clinical studies now echo these findings. From reductions in HbA1c and inflammatory markers to improvements in lipid profiles and insulin sensitivity, the early human evidence is promising. These are not miracle cures—but metabolic nudges that push the body back toward its innate balance, especially when paired with lifestyle realignment.

As we close this chapter, we leave not with finality but with purpose. The story of citrus polyphenols in metabolic reprogramming is still unfolding. While this review focused on their core impact on insulin resistance, oxidative stress, mitochondrial-ER integrity, and inflammatory remodeling, much remains to be explored.

For now, we end where we began: with complexity, with nature, and with a hopeful question. If food is information, and polyphenols are its syntax, then perhaps within the citrus grove lies not just nutrition, but a code for reprogramming metabolism itself. And we have only just started decoding it.

## Figures and Tables

**Table 1:** Citrus polyphenols and metabolic reprogramming in animal and cell studies. The table lists the compound studied, experimental model, intervention characteristics, and major outcomes on insulin signaling, oxidative stress, lipid metabolism, or mitochondrial/ER function.

Compound	Model/System	Intervention Details (Dose and Duration)	Primary Outcomes/Mechanisms	Source
Neohesperidin	HFD-fed mice	50–100 mg/kg/day, 8–12 weeks	↑ AMPK–PGC-1α → ↑ mitochondrial biogenesis, ↓ steatosis	[46]
Nobiletin	HFD-fed mice	100 mg/kg/day, 8 weeks	↑ FA oxidation, ↑ energy expenditure; AMPK-independent	[47]
Nobiletin	Hepatocytes	10–50 μM, 24–48 h	Restores Bmal1 → ↑ lipid/OXPHOS metabolism	[48]
Nobiletin	Insulin-resistant mice	50 mg/kg/day, 6 weeks	↓ VLDL secretion; improved lipid/glucose metabolism	[47]
Nobiletin	HepG2 cells	25 μM, 24 h	↑ PGC1α, CPT1, UCP2 → ↑ β-oxidation	[47]
Nobiletin	ob/ob mice	100 mg/kg/day, 6 weeks	↑ GLUT4, ↑ Akt phosphorylation → improved insulin sensitivity	[49]
Naringenin	MCD or HFD mice	50–100 mg/kg/day, 8–12 weeks	↑ AMPK → ↑ autophagy, ↑ mitochondrial biogenesis	[50]
Naringenin	Hepatocytes/mice	10–50 μM in vitro; 100 mg/kg/day in vivo	↑ AMPK, ↑ ATF3 → ↓ metabolic inflammation	[51]
Naringin	HFD-fed mice	100 mg/kg/day, 10 weeks	↑ AMPK → ↓ SREBP-1c/FAS, ↑ redox balance	[52]
Naringin	Fructose-fed rats	40 mg/kg/day, 8 weeks	↑ Nrf2/HO-1 → antioxidant response; ↓ ChREBP/SREBP-1c	[53]
Naringin	HFD mice	100 mg/kg/day, 12 weeks	↑ TFEB → lipophagy → ↓ hepatic lipid droplets	[54]
Hesperidin	LO2 hepatocytes (HG)	25–100 μM, 24–48 h	↑ ATP, restores ΔΨm via AKT/GSK3β	[55]
Hesperidin	Hyperlipidemic rats	100 mg/kg/day, 6 weeks	↑ SOD, ↑ catalase; preserved mitochondrial enzymes	[56]
Hesperidin	Neurons (hyperglycemia)	25 μM, 24–48 h	Improves ATP/redox; ↓ mitochondrial dysfunction	[57]
Hesperetin	Aging mice	50 mg/kg/day, 8 weeks	↑ Cisd2 expression → maintenance of metabolic health	[58]
Limonene	Mice model	100 mg/kg/day, 6 weeks	↑ mitochondrial respiration, ↓ ROS	[59,60]
Eriocitrin	HFD rats	25–50 mg/kg/day, 8 weeks	↑ mitochondrial biogenesis, ↓ steatosis	[61]
Sudachitin	C57BL/6J, db/db mice	50 mg/kg/day, 8 weeks	↑ β-oxidation, ↑ mitochondrial biogenesis	[62]
Tangeretin	Diabetic rats	100 mg/kg/day, 6 weeks	↑ GLUT4, ↑ antioxidant enzymes	[63]
Naringenin	NAFLD mice	100 mg/kg/day, 10 weeks	↓ NLRP3/NF-κB, ↓ IL-1β → metabolic reprogramming	[64]
Naringenin	NAFLD mice (metabolomics)	100 mg/kg/day, 12 weeks	Modulates gut microbiota → improved host metabolism	[65]
Naringenin	Muscle cells	25–50 μM, 24 h	↑ p-AMPK → ↑ glucose uptake, ↑ mitochondrial content	[66]
Naringin	Hepatocytes, HFD mice	25 μM in vitro; 100 mg/kg/day, 8 weeks	AMPK–IRS1–MAPK pathway → improved insulin signaling	[67]
Naringenin	MASLD mice	100 mg/kg/day, 12 weeks	↑ PPAR, ↑ lipid oxidation, gut microbiota shift	[68]
Naringenin	Mice (aerobic fitness)	100 mg/kg/day, 4 weeks	↑ oxidative fibers, ↑ aerobic metabolism	[69]
Naringin	KK-A(y) mice	100 mg/kg/day, 8 weeks	↑ AMPK → ↓ glucose/lipids, ↑ insulin sensitivity	[70]
Neohesperidin	DIO mice, HepG2 cells	50–100 mg/kg/day, 12 weeks; 25 μM in vitro	↑ FGF21, ↑ AMPK → improved lipid regulation	[71]
Hesperidin	MASLD mice	100 mg/kg/day, 8 weeks	↓ insulin resistance, ↓ oxidative stress	[72]
Nobiletin	HepG2 cells	25 μM, 24 h	↑ AMPK, ↓ lipogenesis	[73]

## Data Availability

Not applicable.

## Abbreviations

AMPK	AMP-activated protein kinase
ALT	Alanine aminotransferase
AST	Aspartate aminotransferase
ATF3	Activating transcription factor 3
BMI	Body mass index
CPT1	Carnitine palmitoyltransferase 1
CRP/hs-CRP	C-reactive protein/high-sensitivity C-reactive protein
ER	Endoplasmic reticulum
FA	Fatty acid
FAS	Fatty acid synthase
FMD	Flow-mediated dilation
FOXO1	Forkhead box protein O1
GGT	Gamma-glutamyl transferase
GLP-1	Glucagon-like peptide-1
GLUT4	Glucose transporter type 4
HbA1c	Glycated hemoglobin A1c
HDL	High-density lipoprotein
HFD	High-fat diet
HMGCR	3-hydroxy-3-methylglutaryl-CoA reductase
HOMA-IR	Homeostatic model assessment of insulin resistance
IL-1β, IL-6	Interleukin-1 beta, Interleukin-6
IRS1	Insulin receptor substrate 1
JNK	c-Jun N-terminal kinase
LDL	Low-density lipoprotein
MAPK	Mitogen-activated protein kinase
MASLD	Metabolic dysfunction–associated steatotic liver disease
MCD	Methionine-choline deficient diet
NF-κB	Nuclear factor kappa-light-chain-enhancer of activated B cells
NLRP3	NOD-, LRR- and pyrin domain-containing protein 3 inflammasome
NO	Nitric oxide
Nrf2	Nuclear factor erythroid 2–related factor 2
OXPHOS	Oxidative phosphorylation
PERK	PKR-like ER kinase
IRE1	Inositol-requiring enzyme 1
ATF6	Activating transcription factor 6
PGC-1α	Peroxisome proliferator-activated receptor gamma coactivator 1-alpha
PI3K/AKT	Phosphoinositide 3-kinase/protein kinase B pathway
PPARγ	Peroxisome proliferator-activated receptor gamma
ROS	Reactive oxygen species
SCFA	Short-chain fatty acid
SOD	Superoxide dismutase
SREBP1c	Sterol regulatory element-binding protein 1c
TFEB	Transcription factor EB
TG/TC	Triglycerides/Total cholesterol
TNF-α	Tumor necrosis factor-alpha
T2DM	Type 2 diabetes mellitus
UPR	Unfolded protein response
UCP2	Uncoupling protein 2
VLDL	Very-low-density lipoprotein
